# Synthesis of Planar Circular Arrays with Quantized Amplitude Weights

**DOI:** 10.3390/s21206939

**Published:** 2021-10-19

**Authors:** Zhiqiu He, Gang Chen

**Affiliations:** Electronic Information School, Wuhan University, Wuhan 430072, China; zqhe1234@whu.edu.cn

**Keywords:** array synthesis, quantized weights, planar circular array, low maximum sidelobe level

## Abstract

A new stepwise and radially processed method for synthesizing uniformly distributed circular planar arrays with quantized weights is proposed in this paper. This method is based on a generalized analytical equation describing that for high directivity focusing arrays, minimizing the weighted mean square error between the reference pattern and the synthesized pattern is equivalent to minimizing the mean square error between the radial cumulative distributions of the reference distribution and the synthesized distribution. This principle has been successfully performed for designing large concentric ring arrays, and in this paper, we extend its use for synthesizing uniformly distributed planar circular arrays with quantized weights. Various numerical examples and comparisons with several reported statistical methods in terms of the lowest Maximum SideLobe Level (MSLL) demonstrate the effectiveness of the proposed method.

## 1. Introduction

Radars with large uniformly distributed array antennas have been widely applied in atmosphere observation [1], military warning, and navigation [2] with an increasing speed. To reduce the kind of the Transmitting/Receiving (T/R) modules to simplify the transmitting feeding network in array antennas as well as to suppress the MSLL as much as possible, several design ideas are presented. First is the thinned arrays. In the previous stage, Willey [3] and Skolnik [4] investigated the density-weighted thinned arrays. Soon afterward, nature-inspired algorithms such as genetic search [5,6] and particle swarm optimization [7] have been exploited to design thinned arrays. However, due to the very intensive computational efforts, those stochastic optimization-based methods are not suited for large array thinning [8]. Later on, the Iterative Fourier Technique (IFT) [8] as well as its derived IFT-based algorithms [9,10] and more recent dynamic programming [11] have been successfully applied to large array thinning. Another solution is the nonuniformly distributed arrays, e.g., [12,13,14,15,16]. Though they may outperform thinned arrays in many aspects theoretically, the interelement spacing is changed, which violates the nature of the uniform distribution.

From the form of excitation coefficients, thinned arrays are two-step [0, 1] quantized weights arrays, and the amplitude coefficients at elements that remained are equal. The primary motivation of array thinning is the reduction in cost and weight. At the same time, a low MSLL can be obtained without evidently widening the Half-Power BeamWidth (HPBW) if the amplitude distributions are properly configured [8]. Though the reported lowest MSLL of thinned arrays has been improved many times, however, the lowest attainable MSLL associates with the number of antennas, when the total number of elements is not large, the MSLL is not low [17]. To further suppress the MSLL without increasing the number of elements, the concept of quantized weights arrays is introduced. Essentially, the distribution generated by quantized weights is a combination of density and amplitude tapering, which further smooths the average amplitude illumination [18]. However, the quantized weights arrays require a set of discrete amplitude weights. Whether the weight set is provided in advance or generated simultaneously during the synthesizing process, extra optimization dimensions are added, thus directly increasing the complexity of solving the problem. In addition, varied methods applied well for synthesizing classical two-step thinned arrays are not applicable here to synthesize quantized weights arrays. Therefore, it is relatively more difficult to synthesize perfect quantized weights arrays.

Till now, only a few studies have addressed this topic [17,19,20,21,22,23]. Even so, they are all implemented in a very similar statistical way. Ball [20] designed several sets of quantized weights and concluded that the set wherein the weights are equally spaced and distributed between zero, and one can obtain one of the largest reductions in MSLL when the selection probabilities are the terms of the binomial distribution. Gao [17] calculated the optimum quantized weights from a nonlinear optimization problem which minimizes the variance between the reference pattern and the synthesized pattern. Except for those, the other used quantized weights are generated randomly [21] or preset with several specific particular values [19,22], which may be far from the global optimum. Li [24] summarized the relationship between the quantized amplitudes arrays with the statistical density tapering arrays and concluded that the latter is only a special form of the former, but the proposed method to design quantized amplitude arrays is essentially the same as that of in [17]. More recently, Shao [22] added some modifications to [21] and found that lower MSLL can be obtained when the illumination probability of one element is also relevant to the illumination probabilities of other elements in the adjacent positions. The performance of [22] is better than that of [21] in some situations but still worse than that of [17].

Except for some theoretical studies mentioned above, quantized weights arrays have been applied to several Very High Frequency (VHF) radars for the observation of neutral atmosphere and Field Aligned Irregularities (FAIs). Unlike the turbulence echo in the neutral atmosphere, the FAIs are also very sensitive to the weak radio frequency signals. In some ionospheric strong scattering events, part of the radar echo may be received by the sidelobes [25]. In this case, the range-time-intensity plot displays the combination of the mainlobe and the sidelobe echoes, which leads to the misjudgment of the actual height and angle of arrival of the FAI echoes. Though the use of the multichannel Spatial Domain Interferometer (SDI) technique can sometimes well reconstruct the actual position of FAIs in space [26]. However, for a routine-operated VHF radar (requiring a long-time operation) to ensure the quality of the data transmission, the number of consecutive pulses in each data block is quite limited. At this time, the SDI performs badly. Therefore, the most reasonable way is to suppress the MSLL of the transmitting beam. As a consequence, some researchers have developed multiple equivalent approaches to meet the lowest SLL requirements. The stationary SOUSY enlarged the interelement spacing at the edge of the array and fed the antennas at the inner positions with higher power [27] simultaneously. The mobile SOUSY adopted a three-level power configuration of [1, 0.5, 0.25] to smooth the current distribution at the transmitting stage [27]. Later, to suppress the MSLL, numerous T/R modules with different powers are adopted in the Indian MST radar to achieve a modified Taylor weighting in both principal directions [28]. Soon, the new Qinzhou MST radar will also consider the quantized weights to guarantee the transmitting beam with a low MSLL [1].

Though the use of quantized weights arrays can be traced back to a very early age, there are still some drawbacks, and there has been no evident progress for a long time. One limitation of the previously reported methods is that it is difficult to select an appropriate reference distribution associated with the lowest MSLL because the selected reference distribution does not show any regularities. Another limitation is that the reported methods cannot achieve a notable and stable MSLL attenuation because there is not a solid theoretical foundation, and final results depend largely on probability. In addition, few studies have found further research on this topic in recent years. The purpose of this paper is to further suppress the MSLL of aperture-limited circular arrays with limited quantized weights as well as to push this work forward. We applied a totally new idea to solve this problem and proposed a new method. The idea was inspired by a synthesis problem of concentric ring arrays. To obtain low MSLL, Milligan [29] developed a technique to design concentric ring arrays. The radius of each ring is specified by dividing the cumulative distribution by the number of rings. Afterward, Bucci [30] exploited an efficient deterministic method for fast-design high-directivity focusing aperiodic concentric ring arrays with multiple constraints along with the increasing demand in satellite applications. This method establishes a generalized analytical equation between the reference array and the synthesized array, revealing that minimizing the weighted mean square error between the reference pattern and the synthesized pattern is equivalent to minimizing the mean square error between the radial cumulative distributions of the reference distribution and the synthesized distribution. A similar approach is also seen in [31]. However, using this kind of method associated with radial cumulative distribution to design aperiodic concentric ring arrays, the pattern consistency of reference array and synthesized array only maintains well at near in sidelobes but not the far ones. Usually, an element pattern is needed to lower the level of the far outside lobes [30].

In this paper, we extend the use of the analytical equation in [30] for synthesizing uniformly distributed circular arrays with quantized weights and low MSLL. Compared with the previously reported methods, the proposed method not only improves the MSLL performance by about 1–2 dB but also obtains the best results of HPBW and array directivity in all test cases with different apertures, which sheds new light on this topic and presents the significance of the proposed work. We organize this paper as follows: In Section 2, we first extend the basic theory [30,31,32] of connecting the patterns and distributions to uniformly distributed circular arrays. In Section 3, we present the proposed method in detail. Various numerical examples and comparisons with several reported statistical methods in terms of the lowest MSLL are given in Section 4, and Section 5 concludes this paper.

## 2. Discussion of the Generalized Relationship Applied for Uniformly Distributed Circular Arrays

For a continuous circular symmetric aperture distribution, the radiation pattern can be expressed as

(1) $$f\left(u\right)=\int_{0}^{R}i\left(\rho \right)\rho J_{0}\left(\beta \rho u\right)d\rho$$

where $J_{0}\left(\cdot \right)$ is the zero-order Bessel function of first kind, *R* is the radius of the circular aperture, and i(ρ) is the normalized amplitude. β=2π/λ with λ being the wavelength. u=sinϑ with ϑ being the angle measured from the boresight.

For uniformly distributed circular arrays, the circular-shaped aperture can be truncated from the square aperture. To preserve some symmetry in the final layout, we assume that one element is exactly at the center of the circular region. Seen from the center element, the array factor of uniformly distributed arrays can be expressed as

(2) $$f_{a}\left(u,\phi \right)=\sum_{n=0}^{N-1}{\tilde{I}}_{n}e^{j\beta r_{n}u\cos\left(\phi -\varphi_{n}\right)}$$

where *N* denotes the total number of the array and ϕ is the azimuth angle describing the field. ${\tilde{I}}_{n}$ and $\varphi_{n}$ represent the illumination and the azimuth of the *n*th element, respectively. $r_{n}$ is the distance between the center element and the *n*th element.

Dividing the whole array by a sequence of equal-width concentric rings, then the array factor (2) can be rewritten as a double summation form

(3) $$f_{a}\left(u,\phi \right)=\sum_{k=1}^{N_{r1}}\sum_{n=0}^{N_{k}-1}{\tilde{I}}_{n}e^{j\beta r_{k,n}u\cos\left(\phi -\varphi_{k,n}\right)}$$

where $N_{r1}$ is the total number of rings and $N_{k}$ is the number of element of the *k*th ring array. Let the width of the concentric rings to a very small value and discard the rings that do not contain any elements; then, in each remained ring, we have

(4) $$\begin{array}{cc} r_{k,n} & \to r_{k}\\ N_{r1} & \to N_{r2} \end{array}$$

where $N_{r2}$ is the number of rings that contain elements. (4) presents that all the radii $r_{k,n}$ in the *k*th ring can be replaced by their mean value $r_{k}$ and the total number of rings reduce to $N_{r2}$. Then, (3) simplifies to

(5) $$f_{a}\left(u,\phi \right)=\sum_{k=1}^{N_{r2}}\sum_{n=0}^{N_{k}-1}{\tilde{I}}_{n}e^{j\beta r_{k}u\cos\left(\phi -\varphi_{k,n}\right)}$$

We can see that (5) describes the array factor of the uniformly distributed circular array in a way similar to the concentric ring array. Introducing the discrete array illumination function in the (ρ,φ) plane

(6) $${\tilde{i}}_{a}\left(\rho ,\varphi \right)=\sum_{k=1}^{N_{r2}}\sum_{n=0}^{N_{k}-1}\frac{\tilde{I}\left(\rho ,\varphi \right)}{\rho }\delta \left(\rho -r_{k}\right)\delta \left(\varphi -\varphi_{n,k}\right)$$

and using the Jacobi–Anger expansion

(7) $$e^{jx\sin\phi }=\sum_{m=-\infty }^{+\infty }J_{m}\left(x\right)e^{jm\phi }$$

(5) can be transformed into

(8) $$f_{a}\left(u,\phi \right)=\int_{0}^{R}J_{0}\left(\beta \rho u\right)\rho d\rho \int_{0}^{2\pi }{\tilde{i}}_{a}\left(\rho ,\varphi \right)d\varphi =f_{a}\left(u\right)$$

where there only remains the zero harmonic for the Bessel functions of the first kind go rapidly to zero when the argument βρu is smaller than the nonzero order, and we focus more on the near-in zenith range (u≪1). Moreover, we can find once the nonzero harmonics are dropped, the array factor $f_{a}\left(u,\phi \right)$ is approximated by an azimuth-independent form $f_{a}\left(u\right)$. Introducing the average radial ring distribution neglecting the azimuthally asymmetric property

(9) $$i_{a}\left(\rho \right)=\int_{0}^{2\pi }{\tilde{i}}_{a}\left(\rho ,\varphi \right)d\varphi$$

and the radial cumulative function

(10) $$\begin{array}{cc} I(\rho ) & =\int_{0}^{\rho }i\left(\eta \right)\eta d\eta \\ I_{a}\left(\rho \right) & =\int_{0}^{\rho }i_{a}\left(\eta \right)\eta d\eta \end{array}$$

and combining (1), (8)–(10) we have [30,31]

(11) $$\frac{f\left(u\right)-f_{a}\left(u\right)}{u}=\int_{0}^{R}\beta \left[I\left(\rho \right)-I_{a}\left(\rho \right)\right]J_{1}\left(\beta \rho u\right)d\rho$$

or applying the Parseval’s theorem [30]

(12) $$\int_{0}^{+\infty }{\left|\frac{f\left(u\right)-f_{a}\left(u\right)}{u}\right|}^{2}du=\beta^{2}\int_{0}^{R}{\left|I\left(\rho \right)-I_{a}\left(\rho \right)\right|}^{2}d\rho$$

provided that the total distributions of reference array and synthesized array are equal

(13) $$I\left(R\right)=I_{a}\left(R\right)$$

where I(ρ) and $I_{a}\left(\rho \right)$ represent the radial cumulative function of the reference distribution and the synthesized distribution, respectively.

## 3. The Proposed Method

Equation (12) has been applied to the synthesis of large concentric ring arrays. In previous work [30], the embedded elements are excited with equal illumination, while the element positions are progressively determined. In this paper, the configuration of the elements is determined in advance. To have a better comparison with the reported work [17,21,22], the used arrays are uniformly distributed circular arrays where elements are arranged into an equilateral square grid with λ/2 spacing. The primary motivation of applying quantized weights is to suppress the MSLL. Then, if a quantized weights set containing $N_{\mathrm{amp}}$ quantized weights is given as

(14) $$a=[a_{1},a_{2},\cdots ,a_{N_{\mathrm{amp}}}]$$

the optimization problem becomes to minimize the MSLL of the synthesized pattern by determining the amplitude at each element that is subject to a constraint that the amplitude should be in the quantized weights set a. Then, the problem can be formulated as

(15) $$\begin{array}{cc} \min_{w} & \mathrm{MSLL}(w)\\ s.t. & w_{i}\in a,i=1,2,\cdots ,N \end{array}$$

where *N* is the total number of the array. MSLL(w) is the MSLL of the synthesized array applying the aperture distribution w. While the used arrays are uniformly distributed, we can fast calculate the array factor through the inverse Fast Fourier Transform (FFT) [8]. Additionally, the interelement spacing of λ/2 ensures that no grating lobes move into the visible space along with the scanning of the main beam. Then, the MSLL can be regarded as the second maxima in the normalized (u,v) plane with u=sinϑcosϕ and v=sinϑsinϕ. Note in this way that both the visible and invisible space are considered when calculating the MSLL. The optimization problem (15) is the general form for synthesizing quantized weights arrays with the lowest MSLL. Moreover, considering the practical realization and reducing the complexity of the feed network as much as possible, the normalized quantized weights set a in descending order and adopted in this paper are preset as [1,0.5,0.25,0]. This amplitude configuration is easy to achieve compared to that in [17] or a sequence of randomly generated weights [21], benefited from the developed Direct Digital frequency Synthesizer (DDS) technique, and we can control the RF power by adjusting the generated power of DDS. All T/R modules only need to work within the saturation amplification region.

The followed subsections present the basic idea of the approach on how to assign the quantized weights a into the aperture distribution w in detail.

### 3.1. Determine the Amplitude Distribution in Each Ring

Since we have known the quantized weights a, the subsequent procedures can be performed similarly to [9,11,32]. We first divide the whole circular aperture by $N_{r}$ concentric circle, with $N_{r}$ being defined as

(16) $$N_{r}=\left\lceil \frac{R}{\Delta r}\right\rceil$$

where ⌈·⌉ represents the ceiling function. The radii vector of all the circles are $r=[r_{1},r_{2},\cdots ,r_{N_{r}}]$ with $r_{N_{r}}$ being the radius of the outermost circle, which equals the aperture radius *R*. Note that the whole aperture is divided into one circle array and $N_{r}-1$ ring arrays. For convenience, all the subarrays are referred to as ring arrays. The followed procedures start at the innermost subarray, and we radially and progressively assign the quantized weights into the synthesized array by approximating the cumulative function of the reference distribution and the synthesized distribution.

There are two criteria before we put the quantized weights into the synthesized array. The most fundamental criterion is that we should try our best to minimize the mean square error of the radial cumulative distribution between the reference distribution and the synthesized distribution within each circle. Another criterion comes from the consideration of (9). To ensure that the amplitudes in each ring are azimuthal symmetric, the selected quantized weights in each ring should be as close as possible and the types of weights should be as few as possible. Therefore, there should be at most two types of weight in each ring, and the two weights should be adjacent in the quantized weights set a. Based on this premise, the most suitable weights and the corresponding numbers in the *k*th ring can be calculated from an optimization problem specified by

(17) $$\begin{array}{cc} \min_{x^{k},i} & \left|c_{\mathrm{syn}}^{r_{k-1}}+a_{i-1}x_{1}^{k}+a_{i}x_{2}^{k}-c_{\mathrm{ref}}^{r_{k}}\right|\\ s.t. & x^{k}={\left[x_{1}^{k},x_{2}^{k}\right]}^{⊤}\in Z^{2}\\ \; & x_{1}^{k}+x_{2}^{k}=N_{e}^{k}\\ \; & 0\leq x_{1}^{k},x_{2}^{k}\leq N_{e}^{k}\\ \; & a_{i}\in a,i=2,3,\cdots ,N_{\mathrm{amp}} \end{array}$$

where $N_{e}^{k}$ is the number of element in the *k*th ring. $x_{1}^{k}$ and $x_{2}^{k}$ are the numbers of weight $a_{i-1}$ and weight $a_{i}$ in the *k*th ring. $c_{\mathrm{syn}}^{r_{k-1}}$ is the cumulative distribution of the synthesized distribution within the radius $r_{k-1}$, and $c_{\mathrm{ref}}^{r_{k}}$ is the cumulative distribution of the reference distribution within the radius $r_{k}$. Note in the first ring, namely k=1, $c_{\mathrm{syn}}^{r_{k-1}}$ equals zero. (17) can be effectively solved from a for-loop, and the subproblem in each for-loop is a typical convex optimization problem.

### 3.2. Place the Quantized Weights to the Ring

Once the most suitable weights and the corresponding numbers in one ring are calculated, the subsequent step is to assign the weights into the elements of the ring reasonably. Considering the premise of (4), the fundamental theoretical derivation weakens the concept of the ring. We emphasize more the sum of the distribution in a narrow ring within a certain radius range. On the other hand, to prevent adverse clustering that further deteriorates the azimuthal symmetric property, the most reasonable way is to space the weights uniformly in the ring [11]. Then, the problem becomes how to space (at most) two kinds of weights into a ring and make the ring satisfy the rotational symmetry property as much as possible. The simplest situation is that there is only one kind of weight to assign, and what we only need is just to assign it to each element in the ring. When there are two kinds of weights in a ring, to ensure the rotational symmetry of the whole ring as much as possible, it can be equivalent to keeping the rotational symmetry of the elements with the same weight in the ring as much as possible. Since the type of weights in one ring is at most two, and the array elements in the ring are divided into two parts according to the weights, i.e., in the *k*th ring, and the number of elements in these two parts are $x_{1}^{k}$ and $x_{2}^{k}$ and $x_{1}^{k}+x_{2}^{k}=N_{e}^{k}$. Define

(18) $$x_{3}^{k}=\min\left(x_{1}^{k},x_{2}^{k}\right)$$

Then, we only need to ensure these $x_{3}^{k}$ elements satisfy the rotational symmetry as much as possible. One modification is applied before we finally start the synthesis. Considering some bad distributions, namely, $x_{3}^{k}$ equals one or two, we replace it with zero or three depending on the requirements of (17) to make it meet (9) as much as possible. Therefore, the number of any kind of weights in one ring should be greater than or equal to three if there are two kinds of weights in that ring.

In addition, it is worth noting that when dividing the whole circular aperture, we only restrict that the elements in each ring do not overlap in the azimuth. However, we do not guarantee that the distances between the elements and the central element are equal and do not guarantee that the elements in one ring have strict azimuth angular periodicity. The above requirements are not completely satisfied even when the ring width Δr is narrow enough. Additionally, when the ring width is quite narrow, the number of elements in each ring is very small, which frequently leads to an extremely nonuniform distribution of the weights. For example, the number of one weight in one ring is only one or two. Moreover, though (4) and (5) suggest that a narrow ring width is recommended. However, after several tests, we notice that when the elements in one ring are not overlapped in azimuth, the final MSLL suppression is not improved with the further decreasing of the width of each ring. To better take into account this contradiction, we take the ring width as half the element spacing (0.25λ) and assume that elements in one ring are equidistant from the center element.

**Figure 1:**
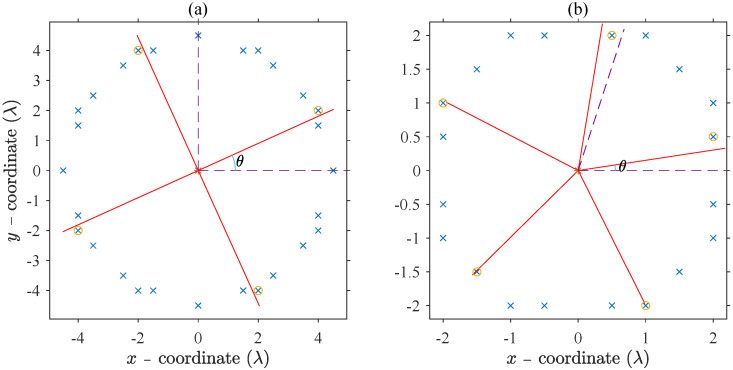
Illustrations on how to select (**a**) 4 and (**b**) 5 elements in one ring to make the part with $x_{3}$ elements satisfies the rotational symmetry as much as possible. The two cases are taken from different rings of one simulation experiment. The blue oblique cross symbols represent the whole elements in the ring, and the ones also surrounded by orange circles denote that the corresponding elements are selected as the part with fewer elements. The red lines coincided with the radius of the ring are uniformly spaced in angle and offset by a random value θ from the azimuth of 0. The θ ranges between 0 and $2\pi /x_{3}$ and is indicated by two purple dotted lines. The number of red lines is also $x_{3}$, and the elements simultaneously marked by the blue oblique cross and orange circle are those that closest to the red lines in azimuth angle. The ∗ symbol is the center element of the circular aperture.

Based on the foregoing presupposition, the initial selection for the $x_{3}$ elements can be obtained from a simple procedure. Figure 1 shows two situations of trying the best to make the $x_{3}$ elements satisfy the rotational symmetry, and the details are described in the caption. The advantage of selecting the elements here in this way is that the elements are not required to be uniformly distributed in the azimuth, and we still can select them out appropriately. The introduction of the random offset vector $\Theta =[\theta_{1},\theta_{2},\cdots ,\theta_{N_{r}}]$ is to avoid the adverse clustering of elements at the azimuth of 0, because in all situations, the azimuth of the red line within the two purple dotted lines starts at the azimuth of 0. On the other hand, the structure of the red lines is strictly azimuthal symmetric; therefore, the random value in any ring, such as in the *k*th ring, $\theta_{k}$ can be limited valued within the range of 0 and $2\pi /x_{3}^{k}$ to decrease the search space.

### 3.3. Refinement

Since a rough quantized weights array can be designed through the previous steps, we can still improve the MSLL performance by a local optimization method. In the previous sections, the offset vector Θ is generated randomly, and it is difficult to achieve global optimum or even local optimum. One possible way is to calculate the optimal Θ directly based on some constraints, but the possibility is strongly limited by the problem size [30]. Therefore, it is unrealistic to optimize the Θ from a global perspective. Inspired from the refinement method mentioned in [11], we propose a local optimization method to optimize the Θ. The process starts at the innermost ring, in each iteration; we only optimize one element of the Θ; for the *j*th iteration, the optimal $\theta_{k}^{j}$ takes the value of the followed optimization problem

(19) $$\begin{array}{cc} \min_{\theta } & \mathrm{MSLL}(\theta_{1}^{j},\cdots ,\theta_{k-1}^{j},\theta ,\theta_{k+1}^{j},\cdots ,\theta_{N_{r}}^{j})\\ s.t. & 0\leq \theta \leq 2\pi /x_{3}^{k} \end{array}$$

where *k* indicates the corresponding index of the ring started at the innermost ring. The iteration number *j* and the ring index *k* have the following relationship:

(20) $$k=[\left(j-1\right)\;\mathrm{mod}\;N_{r}]+1$$

We updated θ one by one along with increasing the number of iterations. To simplify the process, we skipped the iteration if there is only one kind of weight in the corresponding ring, because in this case, whatever value the θ takes, it does not affect the final array layout. The synthesis terminates once the MSLL has not changed in $N_{r}$ iterations or the maximum number of iterations is reached.

Just like (17), the realization of (19) can also be achieved through a for-loop. We can discretize the constrained region $\left[0,2\pi /x_{3}^{k}\right]$ with sufficient dense points. Usually, multiple adjacent points lead to the same situation, and we only need to calculate the MSLL of the same situation once. Thus, the computing time is greatly reduced.

### 3.4. Implementation of the Proposed Method

After much discussion of the details, the proposed method in a single trial can be summarized as follows:

1 Initialize the circular aperture array with specified radius *R* and interelement spacing *d* from a uniformly distributed square array.
2 Choose a suitable reference distribution for the synthesized array.
3 Calculate the amplitude distribution in each ring sequentially.
4 Place the amplitudes to the ring sequentially.
5 Initialize the synthesized array with ten different Θ and keep the case with the lowest MSLL.
6 Employ the refinement process to the remained initial array until the termination conditions are met.

**Figure 2:**
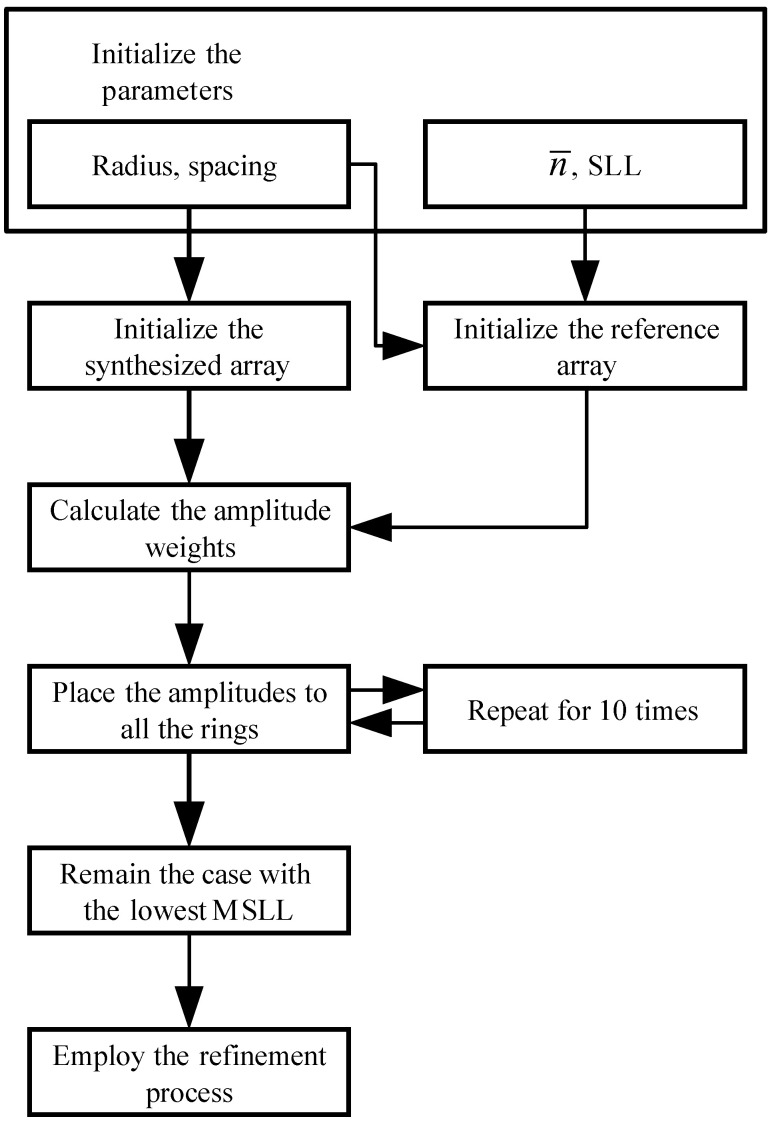
The workflow of the proposed method.

A workflow for illustrating the implementation of the proposed method can refer to Figure 2.

## 4. Numerical Analysis

In this section, we display some typical results generated by the proposed methods and present some comparisons with the reported statistical methods [17,21,22]. The used arrays are circular planar arrays with diameters of 25λ, 33.33λ, and 50λ. The element spacing is 0.5λ, and the width of the ring is 0.25λ for all considered arrays. The proposed method is capable of synthesizing an array with an unlimited number of quantized weights, and in this paper, we only consider the cases with a number of quantized weights of four. Similar to the reported literature [21], the mutual coupling between the elements of the array is not considered in this paper.

### 4.1. Numerical Examples

The reference distribution applied for the proposed method are circular Taylor aperture distributions [33]. Though the circular Taylor distributions are not applicable in some special circumstances, such as facing an incomplete aperture or an oversized interelement spacing [34]. However, in this topic, the arrays we use are uniformly distributed arrays with reasonable interelement spacing, and the sampled results are acceptable.

**Figure 3:**
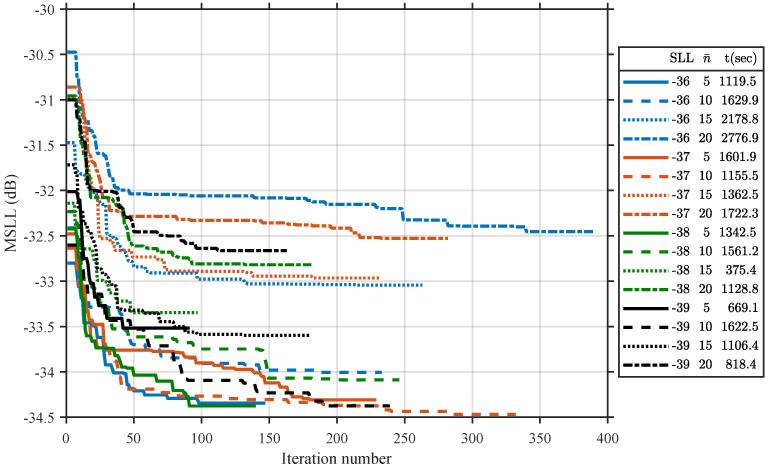
Convergence curves of the MSLL as a function of the iteration number during the refinement process for the quantized amplitudes array with a diameter of 25λ.

We first apply the proposed method to a circular array with a diameter of 25λ in 20 independent trials. Different circular Taylor aperture distributions with SLL range from −36 to −39 dB in a step of −1 dB and $\bar{n}$ (number of equal amplitude sidelobes adjacent to the main beam) ranging from 5 to 25 in a step of 5 are used as reference distributions, respectively. Figure 3 displays the convergence curves of the MSLL as a function of the iteration number during the refinement process of all the trials except the cases with a $\bar{n}$ of 25, because the corresponding performances are quite bad compared to other cases, and to make the figure clearer, we omit this part in the figure. Two conclusions can be drawn from Figure 3. First, the MSLL is heavily dependent on the selected reference distribution, and different selections of reference distribution may lead to an MSLL difference of over 2 dB. Second, $\bar{n}$ seems to play a more important role in final MSLL suppression when $\bar{n}$ is larger than 10—the smaller the $\bar{n}$, the faster the MSLL converges. When $\bar{n}$ is smaller than 10, the convergence curves are jointly controlled by the SLL and $\bar{n}$. The case when the SLL equals −37 dB and $\bar{n}$ equals 10 reaches the lowest MSLL.

**Figure 4:**
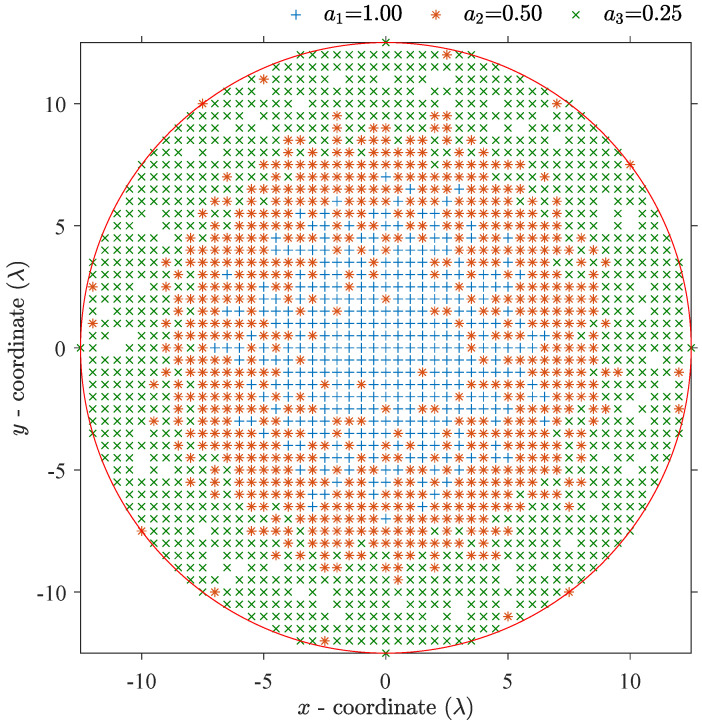
Distribution of the elements of the synthesized array with a diameter of 25λ and a number of quantized weights of four. Different symbols denote that the corresponding elements are illuminated with different amplitudes weights. The vacant positions within the red circle indicate that the corresponding elements are thinned.

**Figure 5:**
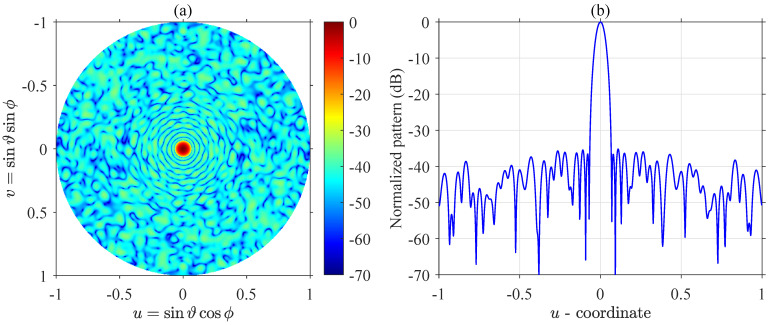
(**a**) The 2-D radiation pattern and (**b**) The *u*-cut plot of the synthesized array present in Figure 4.

**Figure 6:**
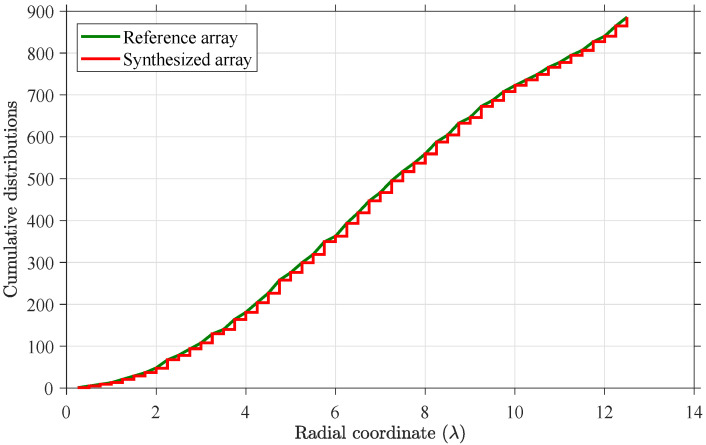
Cumulative distributions as a function of the radial coordinate.

Figure 4 displays the amplitude distribution of the synthesized quantized weights array with the lowest MSLL. The diameter is 25λ, and the number of quantized weights is four. The four quantized weights are [1, 0.50, 0.25, 0], respectively. The MSLL in the whole (u,v) plane is equal to −34.47 dB. Figure 5 displays the surface plot of two-dimensional radiation pattern and the *u*-cut plot of the farfield pattern of the array in Figure 4. From Figure 5, we can notice that the first few sidelobes approximate to −37 dB, and the first few ring-shaped sidelobes near the mainlobe show a circular Taylor-liked pattern because the radial cumulative distributions of reference array and synthesized array presented in Figure 6 coincide well with each other.

The same experiments but for different reference distributions are also carried out for circular arrays with a diameter of 33.33λ and 50λ, both in 20 independent trials. The used SLL of circular Taylor aperture distribution range from −37 to −40 dB and from −41 to −44 dB, both in a step of −1 dB. The used $\bar{n}$ range from 5 to 25 in a step of 5 in all cases. Similar conclusions can also be perceived from the synthesis results. To evaluate the performance of the proposed method, in the next subsection, we present the comparison results between the proposed method and several reported statistical methods.

### 4.2. Comparisons with the Reported Methods

Table 1 summarizes the synthesis results obtained by the proposed method with the lowest MSLL and several reported statistical methods abbreviated as STAT1 [21], STAT2 [17] and ISTAT [22] for circular aperture arrays with diameters of 25λ, 33.33λ, and 50λ. Four quantized weights are adopted in all cases. There are three types of quantized weights, which are denoted as *Fixed*, *Random*, and *Optimized*. The proposed method uses the fixed weights type of [1, 0.5, 0.25, 0], and for a more complete comparison, we also carry the experiments with fixed weights using the STAT and ISTAT. While in the original literature, random weights corresponding to the lowest MSLL are adopted. We also list the experiment results with random weights in Table 1. Unlike other methods, the quantized weights applied in STAT2 are optimized from a nonlinear optimization problem. To have a valid comparison, circular Taylor aperture distribution [4] with SLL ranging from −35 to −60 dB in a step of −1 dB and $\bar{n}$ ranging from 6 to 15 in a step of 1 are applied here for the three statistical methods as the reference distributions. For each distribution, we repeat the experiments 10 times. Thus, each listed result of the three statistical methods is the best outcome in terms of the lowest MSLL in 2600 independent trials. This is also the reason why the adopted reference circular Taylor aperture distributions are different, because the lowest MSLL may correspond to different distribution.

In addition, some modifications are introduced. On the one hand, only the cases analogical to “natural thinning” [4,21] are considered in this paper. On the other hand, in [21], three methods were proposed for designing quantized weights arrays. However, after several trials, we find that nearly in all cases in terms of the lowest MSLL, method 2 obtains better results than method 1 and obtains similar results to those of method 3. Therefore, we only display the results obtained by method 2. Moreover, the ISTAT method in [22] was applied to method 1 of the three methods introduced in [21]. For better results, we also change it to method 2 and reformulate the iteration equations as follows:

(21) $$\begin{array}{cc} F_{n} & =\left\{\begin{array}{cc} a_{k_{2}}, & R_{n}\leq S_{n}\\ a_{k_{2}+1}, & R_{n}>S_{n} \end{array}\right.\\ S_{n} & =\sum_{q=1}^{n-1}\left[\frac{I_{q}-a_{k_{1}}}{a_{k_{1}}-a_{k_{1}+1}}-\frac{F_{q}-a_{k_{1}}}{a_{k_{1}}-a_{k_{1}+1}}\right]+\frac{I_{n}-a_{k_{2}}}{a_{k_{2}}-a_{k_{2}+1}}\\ S_{1} & =\frac{I_{1}-a_{2}}{a_{1}-a_{2}} \end{array}$$

where $I_{n}$ is the amplitude of the *n*th element when the planar reference distribution is expanded to the line array. $S_{n}$ is the density distribution function, and $F_{n}$ is the quantized weight assigned to the *n*th element. $k_{1}$ and $k_{2}$ are the indices of the element in quantized weights set a, which are subject to the constraints of $a_{k_{2}}\geq I_{n}\geq a_{k_{2}+1}$ and $a_{k_{1}}\geq I_{q}\geq a_{k_{1}+1}$.

From Table 1, it can be noted that in all cases, STAT2 obtains lower MSLL than STAT1 and ISTAT, and all MSLL obtained by the proposed method are lower than those obtained by the three statistical methods. Farfield properties including array directivity and HPBW are also presented. The equation for calculating $D_{\max}$ is expressed as [35]

(22) $$D_{\max}=\frac{4\pi |f_{a}\left(\vartheta_{0},\phi_{0}\right){|}^{2}}{\int_{0}^{2\pi }\int_{0}^{\pi /2}{\left|f_{a}\left(\vartheta ,\phi \right)\right|}^{2}\sin\vartheta d\vartheta d\phi }$$

where the main beam points to $\left(\vartheta_{0},\phi_{0}\right)$. In this paper, we let $\vartheta_{0}=0$ and $\phi_{0}=0$. Moreover, to ensure a good numerical accuracy for the calculation of HPBW, we interpolate the original *u*-cut plot data from 1025 to 10,001 points. We can notice that in all cases in Table 1, the proposed method can achieve the narrowest HPBW and the largest array directivity compared to all reported statistical methods.

As to a deeper study of the $\bar{n}$ parameter, other conclusions regarding the HPBW and directivity can be obtained from studying the synthesis results of the circular array with a diameter of 25λ via the proposed method. The HPBW shows a high relationship with the reference taper, which presents an evident negative correlation to the SLL and $\bar{n}$. The array directivity does not show an evident relationship to SLL but seems to be related to $\bar{n}$. For fair comparison issues, in Table 2, we also give the results of the number of the array elements that are “ON” (i.e., with a weight different from 0) of the variety of the methods, and we can notice that the proposed method tends to have a larger fill factor. Further discussions about these issues will be given in a future work.

### 4.3. Discussion of the Proposed Method

Unlike the traditional two-value array thinning, the synthesis problems for quantized weights array are more open. In the proposed method, the final MSLL is associated with multiple variables, including the quantized weights, the reference distribution, the width of the ring sector, and the initial Θ for refinement. In this paper, the dimension of the problem can be reduced through some subjective treatments. Considering the realization, we select a fixed sequence as the quantized weights. As to the ring width, we set it as 0.25λ, because a smaller value does not influence the final results once the elements in one ring are not overlapped in azimuth.

The essence of the proposed method is to approximate the near-in sidelobes of the reference pattern. Thus, it is unlike the reference taper selection strategy revealed in the three reported statistical methods, in which the selected reference distribution does not show any regularities. While the synthesis results show that the SLL of the reference pattern for the proposed method is 3–4 dB lower than the infimum of the MSLL of the array, the infimum of MSLL can refer to the results of the statistical methods. On the other hand, a smaller $\bar{n}$ (≤10) is suggested to ensure a lower MSLL. In addition, the refinement method is not a global optimization method, and only a very limited MSLL suppression of about 2 to 3 dB can be achieved. Therefore, we try to start the refinement process at a low MSLL state as much as possible, and this is the reason why we initialize the Θ for 10 times and select the case with the lowest MSLL before starting the refinement process.

Except for the proposed method, the STAT2 with optimized quantized weights achieve the lowest MSLL, but concerning the other two properties, i.e., maximum directivity and HPBW, the STAT2 does not show any advantage. Moreover, the calculated optimized quantized weights of the STAT2 are hard to practically implement, compared to the fixed-type weights. Thus, in terms of practicability, the proposed method is better.

One evident defect of the proposed method is the required computational time. As shown in the rightmost column of the legend in Figure 3, the simulation time for most cases with a diameter of 25λ is close to half an hour. For cases with larger diameters, the computational time is presented in Table 3. We can notice that the computational time increases exponentially with the increases in aperture. To solve this problem, we can adopt a multiple-process approach, where different reference distributions are assigned to different processors. In this way, a very significant time reduction can be achieved.

## 5. Conclusions

A generalized method to synthesize uniformly distributed circular arrays with quantized weights is described in this paper. The synthesis method requires a model reference distribution and a preset sequence of quantized weights. We first fill the synthesized distribution with quantized weights by making the radial cumulative distribution of the synthesized array as close to that of the reference array as possible. Then, we execute a refinement method to further suppress the MSLL. The essence of the proposed method is to approximate the first few sidelobes of the reference array. Three circular array cases with different diameters ranging from 25λ to 50λ and several quantized weights of four are considered. Detailed numerical examples and comparisons with several reported methods are also presented. In all cases, the proposed method achieves the lowest MSLL, the narrowest HPBW, and the largest array directivity compared to all reported statistical methods, which demonstrates the effectiveness of the proposed method.

## Figures and Tables

**Table 1:** Synthesis results obtained by the proposed method and several reported statistical methods for circular arrays with different diameters.

Circular Array Diameter (λ)	Weights Type	Synthesis Method	Circular Taylor Aperture Distribution		Quantized Weights Set with $a_{1}=1$, $a_{4}=0$		Max. SLL (dB)	Max. Dir. (dBi)	HPBW (Δu)
			**SLL (dB)**	$\bar{n}$	$a_{2}$	$a_{3}$			
25	Fixed	Proposed	−37	10	0.5000	0.2500	−34.4692	33.1488	0.0492
		STAT [17,21]	−41	12			−33.4740	32.5881	0.0512
		ISTAT [22]	−39	9			−33.3035	32.6076	0.0502
	Random	STAT1 [21]	−37	9	0.5542	0.2443	−33.2501	33.1194	0.0496
		ISTAT [22]	−40	6	0.6013	0.2339	−33.0541	32.8283	0.0520
	Optimized	STAT2 [17]	−38	10	0.5919	0.2449	−33.5792	33.2233	0.0498
33.33	Fixed	Proposed	−39	10	0.5000	0.2500	−36.6986	35.5187	0.0376
		STAT [17,21]	−44	14			−35.1273	34.9869	0.0390
		ISTAT [22]	−39	6			−35.1535	35.2634	0.0383
	Random	STAT1 [21]	−43	6	0.5836	0.2344	−34.8121	34.9074	0.0399
		ISTAT [22]	−39	8	0.6825	0.2518	−35.1813	35.3110	0.0505
	Optimized	STAT2 [17]	−42	11	0.5925	0.2473	−35.5330	35.3150	0.0386
50	Fixed	Proposed	−41	10	0.5000	0.2500	−39.2583	38.7858	0.0257
		STAT [17,21]	−44	14			−37.7731	38.6283	0.0261
		ISTAT [22]	−44	15			−37.6708	38.6304	0.0260
	Random	STAT1 [21]	−45	11	0.5668	0.2879	−37.7500	38.4595	0.0266
		ISTAT [22]	−43	10	0.5446	0.1679	−37.9383	38.6416	0.0262
	Optimized	STAT2 [17]	−46	12	0.5880	0.2441	−38.1196	38.4046	0.0267

**Table 2:** Number of the array element that are “ON” (i.e., with a weight different from 0) of the variety of methods presented in Table 1.

**Circular** **Array Diameter** **(λ)**	**Total** **Elements**	**Weights** **Type**	**Synthesis** **Method**	**Number of** **“ON” Elements**
25	1961	Fixed	Proposed	1894
			STAT [17,21]	1750
			ISTAT [22]	1789
		Random	STAT1 [21]	1900
			ISTAT [22]	1677
		Optimized	STAT2 [17]	1751
33.33	3505	Fixed	Proposed	3238
			STAT [17,21]	2951
			ISTAT [22]	3107
		Random	STAT1 [21]	2814
			ISTAT [22]	3171
		Optimized	STAT2 [17]	3055
50	7845	Fixed	Proposed	6912
			STAT [17,21]	6624
			ISTAT [22]	6643
		Random	STAT1 [21]	6051
			ISTAT [22]	7432
		Optimized	STAT2 [17]	6373

**Table 3:** Computational time for the proposed method with the lowest MSLL.

Circular Array Diameter (λ)	Max. SLL (dB)	Number of Trials	Number of Iterations	Computational Time (s)
25	−34.4691	20	332	1155
33.33	−36.6986	20	254	2230
50	−39.2583	20	473	15,862

## Data Availability

All data are available by contacting Zhiqiu He (zqhe1234@whu.edu.cn).

## Abbreviations

The following abbreviations are used in this manuscript:

MSLL	Maximum SideLobe Level
SLL	SideLobe Level
HPBW	Half-Power BeamWidth
STAT1	Statistical method of first version
STAT2	Statistical method of second version
ISTAT	Improved Statistical method
